# Interactions Between Dyspnea and the Brain Processing of Nociceptive Stimuli: Experimental Air Hunger Attenuates Laser-Evoked Brain Potentials in Humans

**DOI:** 10.3389/fphys.2015.00358

**Published:** 2015-12-01

**Authors:** Laurence Dangers, Louis Laviolette, Thomas Similowski, Capucine Morélot-Panzini

**Affiliations:** ^1^Sorbonne Universités, University Pierre et Marie Curie Univ Paris 06, UMR_S 1158 “Neurophysiologie Respiratoire Expérimentale et Clinique”Paris, France; ^2^Institut National de la Santé et de la Recherche Médicale, UMR_S 1158 “Neurophysiologie Respiratoire Expérimentale et Clinique”Paris, France; ^3^Assistance Publique des Hôpitaux de Paris, Groupe Hospitalier Pitié-Salpêtrière Charles Foix, Service de Pneumologie et Réanimation Médicale (Département “R3S”)Paris, France; ^4^Centre de Recherche de l'Institut Universitaire de Cardiologie et de Pneumologie de QuébecQuébec, QC, Canada

**Keywords:** dyspnea, pain, control of breathing, healthy subject, laser-evoked potentials

## Abstract

Dyspnea and pain share several characteristics and certain neural networks and interact with each other. Dyspnea-pain counter-irritation consists of attenuation of preexisting pain by intercurrent dyspnea and has been shown to have neurophysiological correlates in the form of inhibition of the nociceptive spinal reflex RIII and laser-evoked potentials (LEPs). Experimentally induced exertional dyspnea inhibits RIII and LEPs, while “air hunger” dyspnea does not inhibit RIII despite its documented analgesic effects. We hypothesized that air hunger may act centrally and inhibit LEPs. LEPs were obtained in 12 healthy volunteers (age: 21–29) during spontaneous breathing (FB), ventilator-controlled breathing (VC) tailored to FB, after inducing air hunger by increasing the inspired fraction of carbon dioxide -FiCO_2_- (VCCO_2_), and during ventilator-controlled breathing recovery (VCR). VCCO_2_ induced intense dyspnea (visual analog scale = 63% ± 6% of full scale, *p* < 0.001 vs. VC), predominantly of the air hunger type. VC alone reduced the amplitude of the N2-P2 component of LEPs (Δ = 24.0% ± 21.1%, *p* < 0.05, effect-size = 0.74) predominantly through a reduction in P2, and the amplitude of this inhibition was further reduced by inducting air hunger (Δ = 22.6% ± 17.9%, *p* < 0.05, effect-size = 0.53), predominantly through a reduction in N2. Somatosensory-evoked potentials (SEPs) were not affected by VC or VCCO_2_, suggesting that the observed effects are specific to pain transmission. We conclude that air hunger interferes with the cortical mechanisms responsible for the cortical response to painful laser skin stimulation, which provides a neurophysiological substrate to the central nature of its otherwise documented analgesic effects.

## Introduction

Dyspnea (“a subjective experience of breathing discomfort that consists of qualitatively distinct sensations that vary in intensity”) (Parshall et al., 2012) and pain share several characteristics and neural networks (Morelot-Panzini et al., 2007; Von Leupoldt et al., 2009a). This is illustrated by the “dyspnea-pain counter-irritation” phenomenon (Bouvier et al., 2012), namely attenuation of preexisting experimentally induced pain by intervening experimentally induced dyspnea (Stokes et al., 1948; Grönroos and Pertovaara, 1994; Nishino et al., 2008; Yashiro et al., 2011). Different forms of dyspnea are mediated by distinct pathophysiological mechanisms (Lansing et al., 2009). These various forms of dyspnea can have different perceptual effects on pain. For example, air hunger is an adverse respiratory sensation that typically occurs in response to hypercapnia (Parshall et al., 2012). Grönroos and Pertovaara (1994) reported that hypercapnia attenuates the perception of radiant heat and increases the heat pain threshold as well as the ischaemic pain threshold. In the same study conducted in normal humans, hypercapnia did not interfere with electrical pain and mechanically induced pain (Grönroos and Pertovaara, 1994). The sensation of excessive respiratory work/effort is another adverse respiratory sensation that typically occurs in response to mechanical inspiratory constraints (“inspiratory loading”) (Parshall et al., 2012). It is mediated by afferent pathways and central mechanisms different from those involved in air hunger (Lansing et al., 2000). Perceptually, inspiratory loading has been associated with attenuation of thermal pain (Nishino et al., 2008; Yashiro et al., 2011). Neurophysiologically, inspiratory loading inhibits the nociceptive RIII flexion reflex (Morelot-Panzini et al., 2007) and reduces the amplitude of laser-evoked potentials (Bouvier et al., 2012). In contrast, preventing the reflex ventilatory response to hypercapnia (air hunger) does not interfere with the RIII reflex (Morelot-Panzini et al., 2014). In the light of this observation and documented reports of hypercapnia-induced endogenous analgesia (Stokes et al., 1948; Gamble and Milne, 1990; Grönroos and Pertovaara, 1994), we have previously concluded that the analgesia associated with air hunger does not involve spinal mechanisms, but is mediated by central mechanisms (Morelot-Panzini et al., 2014). The present study was designed to test this hypothesis using a laser-evoked potential (LEPs) paradigm. LEPs are elicited by stimulation of cutaneous C-fibers and A∂ fibers. The magnitude of their N1, N2, and P2 components correlates with the intensity of pain perception, although they are strongly influenced by attention and stimulus saliency (Iannetti et al., 2008; Mouraux and Iannetti, 2009). The cortical sources of LEPs include the somatosensory cortex, cingulate gyrus, and anterior insula (Bentley et al., 2001; Garcia-Larrea et al., 2003). The cingulate gyrus (Straus et al., 1997) and insula (Banzett et al., 2000; Peiffer et al., 2001; Von Leupoldt et al., 2008, 2009a) both receive respiratory afferents. The insula plays an important role in dyspnea in general, and more specifically in its affective dimension (Banzett et al., 2000; Peiffer et al., 2001; Von Leupoldt et al., 2008, 2009a). It is considered to play a major role in the pathogenesis of air hunger (Evans et al., 2002; Binks et al., 2014). We therefore hypothesized that air hunger may interfere with brain processing of pulsed laser stimulation of the skin, with corresponding alterations in the characteristics of LEPs. We considered that if this hypothesis were confirmed, it would provide a neurophysiological substrate for the central nature of the analgesic effects of air hunger.

## Materials and methods

### Ethical approval

The study conformed to the standards set by the latest revision of the Declaration of Helsinki, and was approved according to relevant regulations by the “*Comité de Protection des Personnes Ile-de-France VI, Pitié-Salpêtrière*, Paris, France.” The participants received detailed information about the study and provided written consent.

### Participants

Twelve volunteers (age range: 19–30 years; 10 men and 2 women) were recruited to participate in the study. They were naive to physiology and pain experiments. They reported no notable medical history (including chronic or recurrent pain) and declared that they did not suffer from any acute condition or pain at the time of the study. They all underwent medical examination by a physician, who considered them free of any medical condition. The methods used in this study were globally similar to those used in a previous study examining the effects of inspiratory threshold loading on LEPs (Bouvier et al., 2012).

### Experimental conditions

The subjects were instructed to refrain from taking any analgesic and anti-inflammatory medications, alcohol, caffeine, and any psychotropic substances, and to avoid sleep deprivation for 48 h prior to the experiments. During the experiments, the subjects were seated comfortably in a semi-reclined position on an examination chair. Before data acquisition and at 2-min intervals during the study, they were asked to focus their attention on the laser or somatosensory stimulations. Before each laser stimulation, the investigator verbally instructed the subject to concentrate on the oncoming laser pulse.

### Ventilatory measurements

The subjects wore a nose clip and breathed through a mouthpiece connected in series with a pneumotachograph (MLT1000L, AD instruments, Castle Hill, Australia) and a two-way valve (Hans Rudolph 2600 series, KS, USA). Tidal volume (V_T_) was obtained by electrical integration of flow. Minute ventilation (${\text{V}}_{\text{E}}^{\prime }$) was calculated as the product of V_T_ by respiratory rate (f). Inspiratory time (T_I_), expiratory time (T_E_), total cycle time (T_T_), mean inspiratory flow (V_T_/T_I_) and duty cycle (T_I_/T_T_) were derived from the flow signal. End-tidal carbon dioxide tension (PetCO_2_) was measured at the expiratory side of the two-way valve with an infrared CO_2_ analyzer (Servomex 1505, La Plaine Saint-Denis, France). All respiratory signals were recorded by an analog-digital converter (Maclab 16S, Powerlab System, AD Instruments, Castle Hill, Australia; sampling rate 2000 Hz) and Chart™ software (Chart 5.0, AD Instruments, Castle Hill, Australia).

### Dyspnea assessment

During the experiments (see below), dyspnea was assessed in terms of “respiratory discomfort” using a 10 cm visual analog scale (VAS) graded from 0% (“no discomfort”) to 100% (“intolerable discomfort”). This assessment was repeated every minute during each experimental session. At the end of the experiments, the subjects were asked to fill in the “Multidimensional Dyspnea Profile” questionnaire (MDP) (Meek et al., 2012; Banzett et al., 2015) (Table 1), to describe the sensory modalities and the emotions experienced during CO_2_ stimulation.

**Table 1:** **Description of the respiratory sensations and emotions related to breathing during “controlled breathing with CO_2_ stimulation” experiments, assessed according to the multidimensional dyspnea profile (MDP) (Meek et al., 2012; Banzett et al., 2015)**.

**Multidimensional Dyspnea Profile (MDP)**			
	**Median**	**Quartile**	
	**intensity**	**1st**	**3rd**
**BREATHING SENSATIONS**			
My breathing requires muscle work or effort^†^	3	2	5
I am not getting enough air or I am smothering or I feel hunger for air^*^	6.5	5.25	7
My breathing requires mental effort or concentration°	3.5	2	5
My chest and lungs feel tight or constricted^$^	3	0.5	4.75
I am breathing a lot^**^	5	3.5	6
**BREATHING-RELATED EMOTIONS**			
Depressed	0	0	0
Anxious	2	2	3
Frustrated	3	1.25	3
Angry	0	0	0
Afraid	1	0.25	1

* *Ranked 1st by 100% of subjects*.

** *Ranked 2nd by 90% of subjects*.

° *Ranked 3rd by 80% of subjects*.

† *Ranked 4th by 66% of subjects*.

$ *Ranked last by 100% of subjects*.

*Values are medians and 1st and 3rd quartiles*.

### EEG recordings

EEG recordings were performed at Fz, Cz, Pz, C3, C4, T3, T4, A1, and A2, according to the international 10–20 system, using active surface electrodes connected to a V-Amp amplifier (Brain Products GmbH, Gilching, Germany). The electrooculogram was also recorded with Fp1 and Fp2 electrodes placed above both eyes. The EEG signal was sampled at 2 kHz and electrode impedance was maintained at 5 kΩ at all sites during data acquisition. The EEG was recorded and stored on a laptop computer using Brain Vision Recorder software (Brain Products GmbH, Gilching, Germany) and subsequently analyzed with Brain Vision Analyser 2 software (Brain Products GmbH, Gilching, Germany).

### Laser-evoked potentials (LEPs)

#### Stimulation

Laser stimulation was applied perpendicularly to the dorsum of the right hand using a CO_2_ laser stimulator (Neurolas CO_2_ Laser System, Electronic Engineering, Florence, Italy; wavelength: 10.6 μm, intensity: 1.5–15 W, pulse duration adjustable to 10 or 15 ms, beam diameter: 4 mm). Our objective was to deliver laser stimulation close to the A∂ fiber threshold, namely at the intensity eliciting a burning or stinging sensation with a stimulation-sensation delay below 600 ms. This was determined individually, on the day where the actual recordings were performed, and just before starting them, by stepwise increases in the duration and/or power of the laser pulse, with a fixed beam diameter of 4 mm and a fixed distance of 50 cm between the site of stimulation and the laser output lens. A visible light He-Ne pilot laser was used to identify the area to be stimulated. The intensity of the corresponding pain was described in terms of intensity (on a VAS scale ranging from 0% “no pain” to 100% “intolerable pain”), time to onset, duration, and type of sensation (burning or stinging). To reduce the risk of skin burns or erythema and to avoid nociceptor fatigue or sensitization (Greffrath et al., 2007), the site of stimulation was moved by a few millimeters (to an area of naive skin) between each stimulation. In the end, the average stimulation intensity was 6.3 ± 1.2 mJ/mm^2^. During the experiment itself (sequence of respiratory conditions), laser stimulations were delivered with a constant interstimulus interval of 10 s, with each stimulus preceded by a verbal warning of the impending stimulation and instructions to refrain from blinking. Of note, the subjects were not asked to behaviorally rate the sensation elicited by the laser stimulations during the experiments. This was because we felt that it was critical to minimize interferences with the dyspnea behavioral ratings and avoid confusion in a context that was very difficult for the subjects (unpleasant and emotionally challenging respiratory stimulus, repeated reminders that laser pulses were coming and that they had to focus on them, need to concentrate on the dyspnea ratings). This choice was driven by the correlations between LEPs amplitude and pain ratings that have been consistently reported in the literature (Bromm and Treede, 1991; Beydoun et al., 1993; Arendt-Nielsen, 1994; Ohara et al., 2004).

#### Signal processing

The characteristics of the LEPs were studied by processing the EEG signal as follows: definition of an extracephalic reference (linked earlobes A1-A2); band-pass filtering (from 0.5 to 30 Hz); segmentation in 500 ms pre-stimulus to 1500 ms post-stimulus epochs; and baseline correction using a pre-stimulus window (from −500 to 0 ms). Online automatic artifact detection previously rejected all signals with an amplitude greater than ±100 μV and voltage > 65 μV^*^ms^−1^. Sweeps contaminated by electrooculogram artifacts were rejected by visual inspection. Finally, average waveforms were obtained for each subject in each experimental condition. Two main components, N2 and P2, were identified at Cz. N2 was the negative peak occurring 150–300 ms after the onset of the stimulus. P2 was the positive peak with the maximum amplitude that occurred 200–500 ms after stimulus onset. Amplitudes were measured from baseline to peak, and the latencies were defined as the time elapsed between the onset of the component. In line with the literature, the N2-P2 peak to trough amplitude was also measured and considered as a “summarizing feature” of the LEP.

### Somatosensory-evoked potentials

Somatosensory-evoked potentials (SEPs) were studied as a control, to rule out global sensory inhibition and the subject's level of attention as explanations for putative inhibition of LEPs.

#### Stimulation

The right median nerve was stimulated at the wrist using an electrical stimulator (MEB 2200, Nihon Kohden, Tokyo, Japan). The perception threshold was measured using stepwise increases in the intensity of the stimulating current. The intensity of stimulation was then set at 200% of the perception threshold and the stimulation frequency was set at 2 Hz. The average stimulation intensity was 3.16 ± 0.69 mA and the mean number of shocks was 1157.9 ± 67.5.

#### Signal processing

The characteristics of somatosensory-evoked potentials were studied by processing the EEG signal as follows: definition of a new reference (Fz); band-pass filtering (from 30 to 3000 Hz); segmentation in 10 ms pre-stimulus to 40 ms post-stimulus epochs for N20 and P25 components and 10 ms pre-stimulus to 200 ms post-stimulus epochs for the N140 component; and baseline correction using a pre-stimulus window (from −10 to 0 ms). Online automatic artifact detection previously rejected all signals greater than ± 40 μV. Finally, average waveforms were obtained for each subject in each experimental condition. The peak latency and baseline-to-peak amplitude of the somatosensory-evoked potential components were measured in C3-Fz. The N20 component was identified as the negative peak occurring between 15 and 25 ms after stimulus onset and the P25 component was identified as the positive peak with maximum amplitude occurring between 20 and 35 ms after stimulus onset. The N140 component was identified as the negative peak with maximum amplitude in the 130–160 ms time window following the electrical stimulus (Garcia-Larrea et al., 1995). The amplitude of the components was measured from baseline to peak and their latency was measured from the onset of laser stimulation to the peak.

### Ventilator-controlled breathing and experimental air-hunger dyspnea

#### Ventilator-controlled breathing (VC)

This condition served as a control, to unveil possible effects of positive pressure ventilation on LEPs and SEPs, and therefore contribute to the interpretation of a putative effect of the air hunger condition (see below). Subjects were ventilated via a mouthpiece using a Siemens Servo 900C ventilator (Siemens, Solna, Sweden). Respiratory rate, tidal volume, and inspiratory time were adjusted according to the subject's resting breathing pattern and remained constant throughout the experiment. Subjects were instructed to remain passive during mechanical ventilation. The fraction of oxygen in the inspired gas (FiO2) was set at 50% to avoid a contribution of hypoxia to air hunger (Moosavi et al., 2003). The ventilator was set so as to minimize the possibility for the subjects to trigger additional breaths.

#### Ventilator-controlled breathing with CO_2_ stimulation (VCCO_2_)

**Figure 1:**
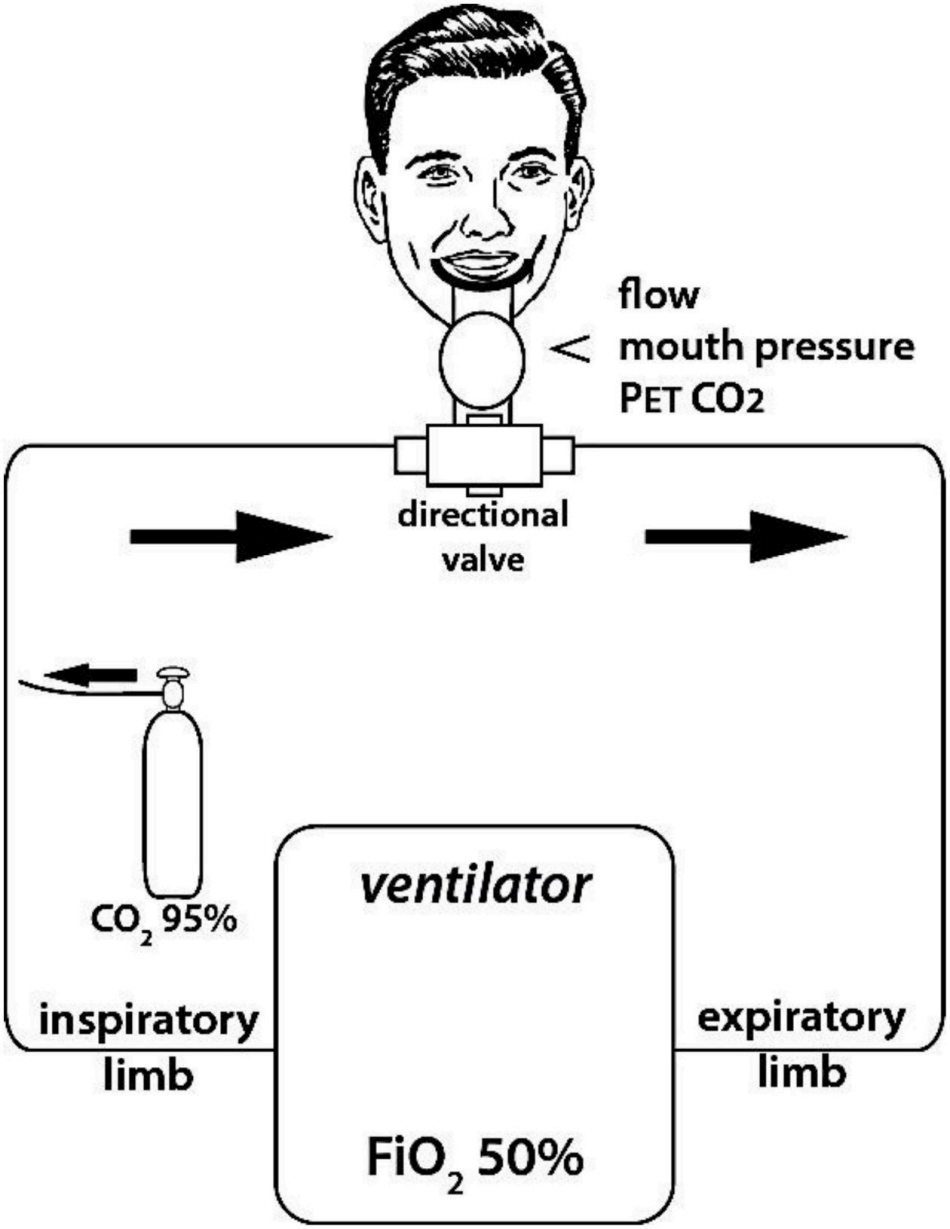
**Experimental set up used to induce experimental dyspnea**. Dyspnea was induced by enriching the inspired gas in CO_2_ (i.e., increasing FiCO_2_) while hindering the ventilatory response to CO_2_ by controlling breathing with a ventilator (fixed tidal volume and respiratory rate, as determined during a preliminary training session to ensure passive ventilation of the subjects to be). FiCO_2_ was fine-tuned in order to maintain respiratory discomfort between 5 and 6 on a 10 cm “respiratory discomfort” visual analog scale (VAS). Flow, mouth pressure, and PetCO_2_ were monitored continuously during the session.

To induce air hunger, 95% CO_2_ was instilled into the inspiratory limb of the breathing circuit to increase the inspired fraction in CO_2_ (FiCO_2_) (Figure 1). The quantity of CO_2_ so administered was taylored on both PetCO_2_ (either maintained or increased, but never allowed to decrease) and on the degree of respiratory discomfort rated by the subjects that was maintained between 50 and 60% of the full dyspnea VAS scale. It ensues that the CO_2_ content of the inspired mixture varied between subjects and with time in a given subject.

#### Ventilator-controlled breathing recovery (VCR)

After the VCCO_2_ period, the subjects were recorded during ventilator—controlled breathing without CO_2_ stimulation in order to explore recovery.

Of note, prior to the experimental sessions, the subjects were familiarized with ventilator-controlled breathing and ventilator-controlled breathing with CO_2_ stimulation in order to determine adequate settings and eliminate the influence of “discovery” on the subsequent results.

Of note also, the VC-VCCO_2_ sequence was neither counterbalanced nor randomized, VC always being studied before VCCO_2_ (see “Methodological Considerations” under “Discussion”).

### Summary of experimental protocol (Figure 2)

The actual experiments were conducted on a day distinct from the “familiarization” day). After subject preparation (EEG setup, ventilatory measurement calibration, and setup), nociceptive laser-evoked potentials (LEPs) were first calibrated in duration and intensity, and then recorded during four 10-min periods separated from each other by less than 2 min, in the following sequence: (1) spontaneous breathing, FB; (2) ventilator-controlled breathing, VC (see above); (3) ventilator-controlled breathing with CO_2_ stimulation, VCCO_2_ (see above); (4) ventilator-controlled breathing recovery (VCR), after CO_2_ removal. During this sequence, ventilatory variables and PetCO_2_ were continuously monitored, and dyspnea assessments were repeated every minute. At the end of this “LEP” sequence, a 20-min rest was allowed, and the sequence was repeated with non-nociceptive somatosensory-evoked potentials (SEPs) (calibration of the stimulus followed by the same four recording periods). The subjects were then asked to answer the MDP questionnaire.

**Figure 2:**
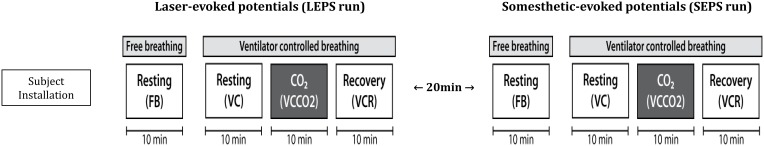
**Experimental sequence**. FB, free spontaneous breathing; VC, controlled breathing (ventilator); VCCO_2_, controlled breathing with CO_2_ stimulation; VCR, controlled breathing after removal of CO_2_ stimulation. Respiratory discomfort was assessed using a 10 cm visual analog scale (VAS) every minute during each of the 10-min experimental sessions.

### Statistics

Statistical analyses were performed using SPSS 18.0 (IBM, USA) and Prism version 5.0 (Graphpad software Inc, CA, USA). The nature of the data distribution was assessed using the Shapiro-Wilk test. LEP-P2 latency, T_E_/T_T_, T_I_/T_T_, and PetCO_2_ were not normally distributed, but LEP-P2 latency had a normal distribution after logarithmic transformation. Normal data sets were described in terms of their mean and standard deviation, whereas non-normal data sets were described in terms of their median and 95% confidence intervals (95%CI). The effects of CO_2_ stimulation on the discrete variables describing breathing pattern (V_T_, T_I_, T_E_, T_T_, V_T_/T_I_, T_I_/T_T_), respiratory discomfort, and scalp potentials were analyzed by Two-way analysis of variance (ANOVA) for repeated measures followed by Tukey *post-hoc* test (normal data) or Friedman's test followed by Dunn's *post-hoc* test (non-normal data). Effect-size was estimated by Cohen's d coefficient. Comparisons were considered statistically significant when the probability p of a type I error was less than 5%.

## Results

### Ventilatory pattern

During the LEP run, PetCO_2_ did not exhibit significant differences between FB, VC, and VCR, but, expectedly, it rose significantly from VC to VCCO_2_ (34.2 ± 2.5 mmHg to 51.3 ± 2.6 mmHg, *p* < 0.001). Likewise, minute ventilation (${\text{V}}_{\text{E}}^{\prime }$) (ANOVA *F* = 3.93; *p* = 0.002), V_T_ (ANOVA *F* = 14.03; *p* < 0.0001), V_T_/T_I_ (ANOVA *F* = 3.84; *p* = 0.02) and T_T_ (*F* = 16.2; *p* < 0.0001) varied significantly across conditions, pairwise comparisons showing that the increases also occurred between VC and VCCO_2_ V_T_ (V_T_ from 1048 ml ± 222 ml to 1355 ml ± 316 ml, *p* < 0.05; ${\text{V}}_{\text{E}}^{\prime }$ from 12.1 l/min ±1.73 l/min to 15.9 l/min ± 2.36 l/min, *p* < 0.05; V_T_/T_I_ form 2.3 l/s ± 0.7 to 3.0 l/s ± 0.7, *p* < 0.05). No significant changes were noted for T_I_/T_T_ (*p* = 0.70) and T_e_/T_T_ (*p* = 0.60).

The same pattern was observed during the SEP runs. Comparison between the LEP and SEP runs did not show significant differences.

### Dyspnea

The subjects did not report any respiratory discomfort during VC, but increasing FiCO_2_ while impeding the ventilatory response with the ventilator (VCCO_2_) induced respiratory discomfort (VAS = 63 ± 6%, *p* < 0.001 vs. VC). According to the MDP questionnaire, the subjects mainly characterized their respiratory sensation as “air hunger” (Table 1). The emotional response was moderate, with the highest score reported for the “frustration” item (Table 1). MDP assessments were similar during FB and VC. No significant difference in dyspnea intensity was observed between the LEP and SEP runs (*p* = 0.65).

### Laser-evoked potentials

**Figure 3:**
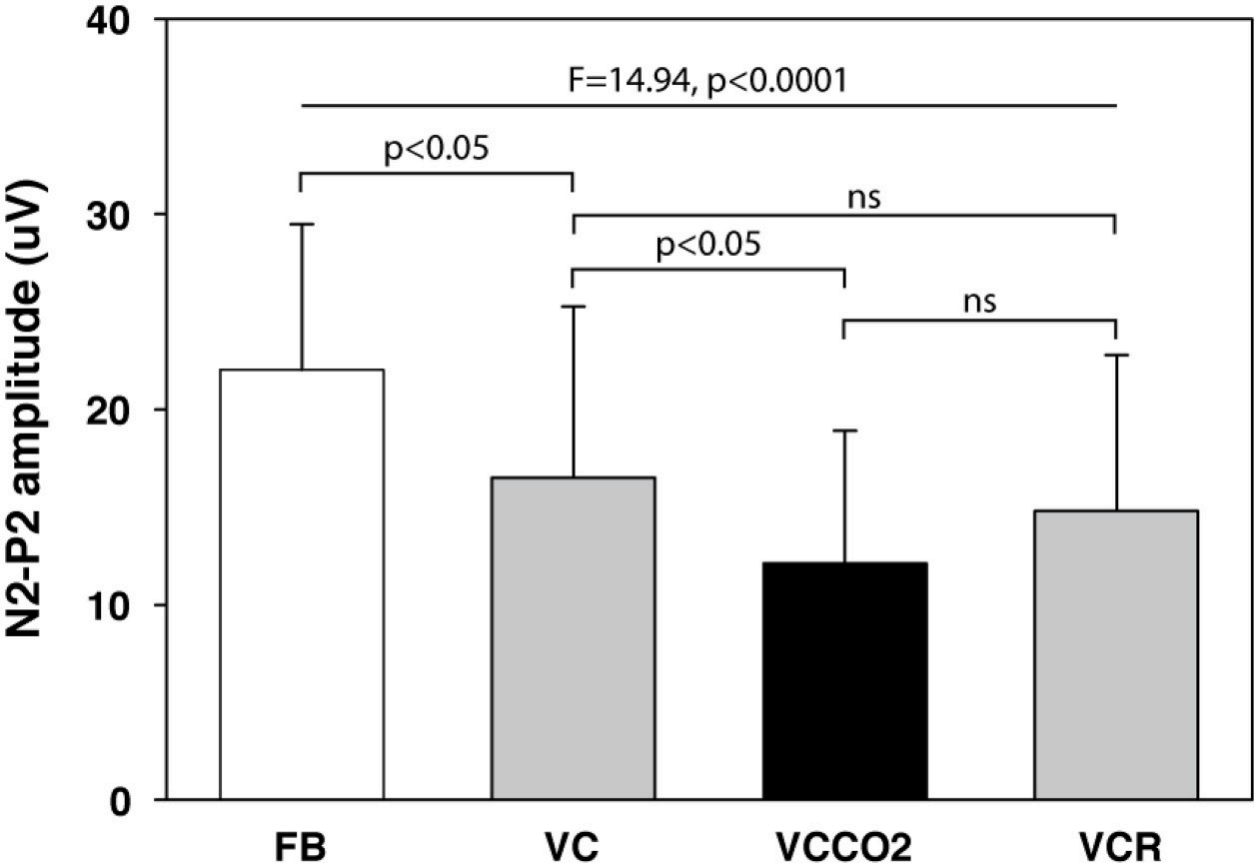
**Amplitude of the N2-P2 component of laser-evoked potentials (12 subjects)**. FB, free spontaneous breathing; VC, controlled breathing (ventilator); VCCO_2_, controlled breathing with CO_2_ stimulation; VCR, controlled breathing after removal of CO_2_ stimulation. Bars depict mean values, with indication of 1 standard deviation.

**Figure 4:**
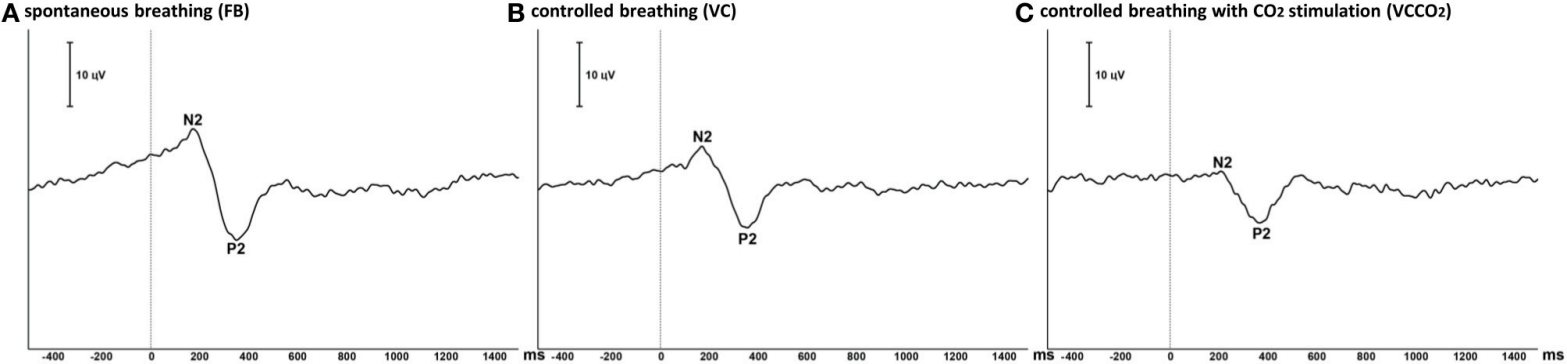
**Grand average of laser-evoked potentials (12 subjects)**. The traces represent the ensemble averaging of the laser-evoked potentials recorded at the vertex (Cz derivation) in the 12 participating subjects during free spontaneous breathing **(A)**, controlled breathing **(B)**, and controlled breathing with CO_2_ stimulation **(C)**. Polarity is negative up. Vertical line illustrates the time of laser stimulation.

The pain evaluated by the subjects at the laser perception threshold was 25 ± 13% of full scale on the pain VAS. Table 2 summarizes the LEPs amplitudes and latencies for all experimental conditions. A statistically significant attenuation of N2-P2 amplitude was observed during VC compared to FB (Δ = 24.0% ± 21.1%, *p* < 0.05, effect-size = 0.74) (Figures 3, 4). Further attenuation occurred during the VCCO_2_ condition (VC vs. VCCO_2_: Δ = 22.6% ± 17.9%, *p* < 0.05, effect-size = 0.53) (Figures 3, 4). Of note, the VC-associated reduction in N2-P2 was driven by reduction in P2 without significant change in N2, while the VCCO_2_-associated further reduction in N2-P2 was driven by a reduction in N2 without significant change in P2. We observed a trend to recovery between the VCCO_2_ and VCR conditions but not significant (VCCO_2_ vs. VCR: Δ = 32% ± 53%, ns effect-size = 0.36).

**Table 2:** **Characteristics of the N2-P2 component of laser-evoked potentials according to experimental conditions**.

	**Laser-evoked potentials (LEP)**				
	**N2**		**P2**		**N2-P2**
	**Amplitude (μV)**	**Latency (ms)**	**Amplitude (μV)**	**Latency (ms)**	**Amplitude (μV)**
**SPONTANEOUS BREATHING**					
Baseline (FB) *n* = 12	−9.1 (5.6)	192.3 (43.6)	12.9 (4.7)	356.6 [312.8–406.5]	22.0 (7.5)
**CONTROLLED BREATHING**					
Baseline (VC) *n* = 12	−7.4 (5.1)	195.2 (60.3)	9.05 (5.0)	354.8 [318.6–395.0]	16.5 (8.8)
CO_2_ (VCCO_2_) *n* = 12	−3.6 (5.8)	226.7 (58.1)	8.5 (4.0)	362.8 (67.7)	12.1 (6.8)
Recovery (VCR) *n* = 12	−6.2 (3.5)	181 (29.8)	8.6 (6)	340 [308.5–335.5]	14.8 (8)
Repeated measures ANOVA	*F* = 11.44 *p* < 0.0001	*F* = 2.90 *p* = 0.049	*F* = 4.90 *p* = 0.006	*F* = 0.35 *p* = 0.7885	*F* = 14.2 *p* < 0.0001
***POST-HOC*** **CONTRASTS (*****P*****-VALUES)**					
*FB vs. VC*	ns		< 0.05		< 0.05
*VC vs. VCCO_2_*	< 0.05		ns		< 0.05
*VCCO_2_ vs. VCR*	ns		ns		ns
*FB vs. VCCO_2_*	< 0.05		< 0.05		< 0.05
*VC vs. VCR*	ns		ns		ns
*FB vs. VCR*	ns		< 0.05		< 0.05

*Values are means (SD) or medians [95% CI] depending on distribution*.

### Somatosensory-evoked potentials

The average intensity of electrical stimulation used for SEPs was 3.16 ± 0.69 mA. Absolute values for amplitude and latency of SEP components N20 and P25 are shown in Table 3. The amplitude of N20-P25 components (*p* = 0.87), the latency of the N20 component (*p* = 0.26) and the latency of the P25 component (*p* = 0.35) did not vary significantly across conditions. The amplitudes and latencies of N140 did not vary significantly across conditions (*p* = 0.35 and *p* = 0.72, respectively).

**Table 3:** **Characteristics of somatosensory-evoked potentials according to experimental conditions**.

	**Somatosensory-evoked potentials (SEP)**					
	**N20**		**P25**		**N140**	
	**Amplitude (vs)**	**Latency (ms)**	**Amplitude (μV)**	**Latency (ms)**	**Amplitude (μV)**	**Latency (ms)**
**SPONTANEOUS BREATHING**						
Baseline (FB) *n* = 12	−0.60 (0.5)	18.9 (1.1)	3.7 (1.8)	29.4 (7.1)	−1.7 (0.9)	141.5 (12)
**CONTROLLED BREATHING**						
Baseline (VC) *n* = 12	−0.47 (0.50)	18.5 (1.6)	3.6 (1.5)	29.5 (6.8)	−1.8 (1.0)	140.0 (12.3)
CO_2_ (VCCO_2_) *n* = 12	−0.70 (0.70)	18.0 (1.6)	3.5 (2.0)	29.0 (6.5)	−1.7 (0.7)	138.5 (11.0)
Repeated measures ANOVA	*F* = 2.28 *P* = 0.13	*F* = 1.15 *P* = 0.26	*F* = 0.13 *P* = 0.88	*F* = 1.12 *P* = 0.35	*F* = 0.24 *P* = 0.79	*F* = 0.33 *P* = 0.73

*Values are means (SD)*.

## Discussion

In the present study, as expected, exposing normal individuals to CO_2_ and hindering their ventilatory response (VCCO_2_ condition) induced respiratory discomfort that was mainly described as air hunger. This was associated with a significant reduction of the amplitude of the N2-P2 component of the LEPs as compared with the relevant control condition (VC condition).

### Methodological considerations

#### Experimental model

To evaluate the effects of air hunger on LEPs and SEPs, the experimental model had to allow easily adjustable and stable air hunger in order to achieve a compromise between a sufficient intensity of respiratory discomfort, while allowing a 10-min tolerance span. We also had to avoid asking the subjects to concentrate on their breathing, because of the effects of attention on LEPs (Iannetti et al., 2008; Mouraux and Iannetti, 2009). The best way to achieve these objectives appeared to be CO_2_ stimulation combined with “passive” prevention of the ventilatory response, i.e., controlled mechanical ventilation (Banzett, 1996). However, despite the use of controlled mechanical ventilation, the subjects failed to remain passive and were able to significantly increase their V_T_ during the VCCO_2_ condition relative to the VC condition, probably by taking advantage of leaks around the face mask. This issue was addressed by real-time adjustment of FiCO_2_ to maintain a constant PetCO_2_. This experimental model differs from that used in our previous study on the effects of air hunger on the RIII reflex (Morelot-Panzini et al., 2014), in which we asked the subjects to voluntarily restrain their ventilatory response to CO_2_.

#### Interference with headache

Acute hypercapnia causes headache in some individuals, which could be a source of counter-irritation independent of hypercapnia-related air hunger. Subjects were therefore asked about the presence of headache at the end of the training and measurement sessions and negative answers were consistently obtained.

#### Order effect

We chose not to counterbalance or randomize the VC-VCCO_2_ sequence, VC being always performed before VCCO_2_. This choice was made because we did expect VCCO_2_ to reduce the amplitude of the LEPs, and did suspect that this effect could exhibit a certain remanence as in the case of the LEP inhibition induced by dyspnea of the work/effort type (Bouvier et al., 2012). This would have made the interpretation of the VC condition as a control difficult. We acknowledge that this can be viewed as a limitation to the interpretation of our results. However, we do not think that an order effect is intrinsically sufficient to explain our observations, because VCCO_2_ was associated with a N2P2 inhibition that was not only significantly greater than the inhibition seen with VC but also due to different impacts on the individual P2 and N2 components (Table 2, and see below).

### General LEP methodological considerations

It is also important to re-emphasize (see discussion in Bouvier et al., 2012) that the reduction of LEP N2-P2 amplitude in response to a conditioning stimulus (in this case experimental air hunger) must be interpreted cautiously, as it can be induced by negative modulation of nociceptive transmission. For example, LEPs decrease over time in response to repeated stimulations (Weiss et al., 1997). We tried to limit the impact of this type of habituation by changing the site of stimulation between each stimulus. Most importantly, LEPs are sensitive to attention (Plaghki et al., 1994; Lorenz and Garcia-Larrea, 2003) and are closely correlated with attentional reorientation (Mouraux and Iannetti, 2009). Attentional reorientation could therefore explain attenuation of LEPs observed in response to application of mechanical ventilation and then in response to experimental air hunger (Figures 3, 4). We tried to control for this attentional influence by warning our subjects before each laser stimulation and by asking them to focus on the skin sensation. This was also a reason for not asking the subjects to rate the laser-evoked pain behaviorally (see below). Of note, as in our study on the effects of inspiratory threshold loading on LEPs (Bouvier et al., 2012), the N140 component of SEPs was not influenced by the experimental conditions (Table 3). As N140 is sensitive to attentional factors (Garcia-Larrea et al., 1995; Eimer and Forster, 2003), the absence of change during controlled mechanical ventilation and experimentally induced air hunger can be considered to be an argument against a major effect of attentional modulation on our results.

Of note, we did not behaviorally assess the perception of the pain induced by laser stimuli during the experiments (see reasons in “Materials and Methods”). We therefore acknowledge that we cannot be certain that the effects of hypercapnia and air hunger on subjective pain perception that have been reported before (for example Grönroos and Pertovaara, 1994) were actually present in our subjects. Nevertheless, this not an unreasonable assumption, insofar as correlations between the subjective perception of pain and the amplitude of the LEPs have consistently been established in the literature (Bromm and Treede, 1991; Beydoun et al., 1993; Arendt-Nielsen, 1994; Ohara et al., 2004). At any rate, observing a LEP inhibition during the experimental induction of air hunger, as we did, suffices to support the notion that air hunger can interfere with the brain processing of noxious stimuli.

### Inhibitory effect of mechanical ventilation

Mechanical ventilation alone (VC period) induced inhibition of LEPs irrespective of respiratory discomfort, which is an unexpected and novel finding. Mechanical ventilation has a non-chemical inhibitory effect on inspiratory activity (Fauroux et al., 1998) and depresses the excitability of the corticospinal pathway to the diaphragm (Sharshar et al., 2004; Hopkinson et al., 2012), but this would appear to be the first study to suggest a possible inhibitory effect of mechanical ventilation on a sensory pathway. Of note, we did not observe any significant changes in the characteristics of the SEPs during mechanical ventilation (Table 3), suggesting that it did not interfere with the function of the posterior column-medial lemniscus pathway. The observed effects were therefore specific to LEPs. Apart from attentional reorientation (see above), several mechanisms could putatively explain these effects. Mechanical ventilation during the VC period of our experiments was adjusted in such a way as to keep tidal volume, minute ventilation and PetCO_2_ similar to their values during the FB period. The major difference between FB and VC was therefore the inspiratory-related intrathoracic pressure regimen that changed from negative during FB to positive during VC. This change could have triggered stimulation of lung or airway receptors sensitive to mechanical stimuli (review in Kappagoda and Ravi, 2006). Such a stimulation could also be the consequence of changes in respiratory mechanics -lung compliance- induced by the fixed pattern of breathing associated with controlled mechanical ventilation, as opposed to the variable pattern of breathing associated with spontaneous ventilation (Mutch et al., 2000, 2007). Certain lung and airway receptors are mediated by C and a∂ fibers (Undem and Carr, 2001; Undem et al., 2002): their activation could trigger diffuse nociceptive inhibitory controls. Alternatively, the change in the inspiratory-related intrathoracic pressure regimen could have modified the vagal afferent traffic to the brain via stimulation of slowly adapting pulmonary stretch receptors (SARs). The effect of mechanical ventilation on LEPs could then speculatively have been due to respiratory vagal projections to the limbic cortex (see in monkey, Radna and MacLean, 1981) (see also in rats, Aleksandrov et al., 2009). Finally, mechanical ventilation also inevitably increased baseline sensory gating of respiratory afferents in our subjects. According to current concepts on sensory gating in general and respiratory sensory gating in particular, this would correspond to modified thalamic and/or hippocampal activities (Davenport and Vovk, 2009). The thalamus and hippocampus are involved in the brain response to painful laser skin stimulation (Kobayashi et al., 2009; Liu et al., 2011) and might be part of the neural matrix responsible for LEPs. It is therefore theoretically conceivable that modifying thalamic and/or hippocampal activities by applying mechanical ventilation could interfere with LEP generation. A first step toward verifying this theory would be to study the effect of isocapnic mechanical ventilation on respiratory sensory gating, e.g., by studying the effect of mechanical ventilation on the cortical response to repeated inspiratory occlusions (Chan and Davenport, 2008). Finally, VC alone could have induced emotional changes even in the absence of dyspnea, which could in turn have had an impact on LEPs. Our experimental design made it impossible to look for such changes (e.g., by using the MDP questionnaire) because the VC and VCCO_2_ conditions were studied in immediate sequence.

### Air hunger and LEP inhibition

Experimentally induced respiratory discomfort, predominantly consisting of air hunger, further reduced inhibition of LEP N2-P2 as compared to the controlled breathing condition, which is consistent with the general notion that dyspnea can inhibit nociception, and with the documented analgesic effect of hypercapnia (Grönroos and Pertovaara, 1994). We have previously shown that a similar -although not identical, see above- experimental paradigm did not result in inhibition of the spinal RIII nociceptive reflex (Morelot-Panzini et al., 2014). Together with the fact that hypercapnia is unlikely to stimulate C-fibers in normal humans (see discussion in Morelot-Panzini et al., 2014), “classical” counter-irritation (activation of descending nociceptive inhibitory controls by heterotopic noxious stimulation) is therefore an unlikely explanation for the LEP inhibition observed during the VCCO_2_ part of our protocol. This conclusion contrasts with that of the study of inhibition of laser-evoked potentials associated with the dyspnea induced by inspiratory threshold loading (Bouvier et al., 2012), further supporting the concept that air hunger and the sensation of excessive inspiratory work/effort are not mediated by the same pathways (see Parshall et al., 2012). Hypercapnia-related analgesia involves endogenous opioids in rats (Gamble and Milne, 1990) and experiments designed to induce dyspnea in humans can increase endorphin production (Akiyama et al., 1993). Insofar as LEPs are modulated by opioids (Truini et al., 2010; Hoeben et al., 2012), our observations may be related to air hunger-induced endorphin production. Finally, attenuation of LEPs by air hunger could be due to “competition” at the cortical level. LEPs in response to noxious laser skin stimulation denote activation of a complex network comprising several cortical and subcortical structures including the thalamus, anterior insula, prefrontal cortex, anterior cingulate cortex, and secondary somatosensory cortex (Garcia-Larrea et al., 2003; Veldhuijzen et al., 2009). Several of these structures are involved in the pathogenesis of respiratory sensations (Davenport and Vovk, 2009) and dyspnea (Von Leupoldt et al., 2009a). In this view, it is interesting to note that the N2-P2 reductions that we observed during VC and VCCO_2_ probably did not proceed from the same mechanisms. Indeed, Table 2 indicates that VC impacted N2-P2 mostly through reductions in P2, while air hunger (VCCO_2_) impacted N2-P2 mostly through reductions in N2. There are arguments in the literature suggesting that N2 and P2 represent different regional contributions to the laser-evoked response. Likewise, and to put thing very simply, P2 has been strongly associated with activation of the anterior cingulate cortex (ACC), while N2 has been associated, albeit less strongly, with activation of the insula (Garcia-Larrea et al., 2003; briefly reviewed in Cruccu et al., 2008; see also Veldhuijzen et al., 2009). Yet the insula is strongly and consistently activated during air hunger induction paradigms while this is less the case regarding the ACC (Banzett, 1996; Liotti et al., 2001; Evans et al., 2002). It could thus be postulated that the LEP impact of controlled mechanical ventilation mostly proceeds from a more “sensory” type of mechanism (see above) and that the LEP impact of air hunger mostly proceeds from a more “emotional” type of mechanism. This is however highly speculative and would need both corroboration and specific explanatory experimental designs.

All in all, it seems safe to conclude from our observations that dyspnea of the “air hunger” type interferes with the brain processing of nociceptive stimuli, as globally illustrated by the corresponding reduction in N2-P2 amplitude. Downregulation of insular cortex responses to both dyspnea and pain have been described in patients with asthma (Von Leupoldt et al., 2009b). These findings suggest that the dyspnea-related inhibition of LEPs observed in our subjects could be due to interference at the insular level. Again, specific experiments would be needed to test this hypothesis, by studying the brain functional response to laser skin stimulation and to a constrained ventilatory response to hypercapnia, and their interactions.

### Summary of available dyspnea-pain counter-irritation neurophysiological data and therapeutic inferences

In addition to various studies comparing dyspnea, pain, and their interactions from a perceptual perspective (Stokes et al., 1948; Grönroos and Pertovaara, 1994; Nishino et al., 2008, 2010; Nishino, 2011; Yashiro et al., 2011), four previous studies have investigated the effects of experimentally induced dyspnea on either the RIII spinal reflex (Grönroos and Pertovaara, 1994; Morelot-Panzini et al., 2007, 2014) or LEPs (Bouvier et al., 2012). Inspiratory threshold loading, mainly associated with the “excessive work/effort” type of dyspnea, inhibits the RIII reflex (Morelot-Panzini et al., 2007). It also inhibits LEPs (Bouvier et al., 2012), with a relationship between the intensity of dyspnea induced and the magnitude of inhibition. Hypercapnia (Grönroos and Pertovaara, 1994) and prevention of the ventilatory response to CO_2_, associated with the “air hunger” type of dyspnea, does not inhibit the RIII reflex (Grönroos and Pertovaara, 1994; Morelot-Panzini et al., 2014). As previously discussed (see Morelot-Panzini et al., 2014), these combined observations indicate that “excessive work/effort” and “air hunger” interfere with pain via different mechanisms. They suggest that, in experiments conducted on healthy subjects, dyspnea-pain counter-irritation is mediated by both “peripheral” and “central” mechanisms in the case of “excessive work/effort” dyspnea, while “peripheral” mechanisms are not involved in the case of air hunger. This is consistent with the pivotal role of C-fiber stimulation in RIII inhibition and the lack of implication of C-fibers in the response to CO_2_ (Coleridge et al., 1978; Lin et al., 2005; reviewed in Morelot-Panzini et al., 2014). The present study provides further evidence by strongly suggesting that air hunger interferes with the cortical mechanisms responsible for the cortical response to painful laser skin stimulation. However, we acknowledge that the respiratory discomfort experienced by our subjects, although dominated by “air hunger,” was multimodal (Table 1). From the perspective of future pharmacological approaches to the treatment of dyspnea and, more specifically, targeting non-opioid mechanisms to alleviate respiratory discomfort (e.g., Mahler et al., 2014), the available evidence suggests that substances interfering with C-fibers should be more active on the “work/effort” component of dyspnea and substances with a central nervous system target should be more active on the “air hunger” component of dyspnea.

## Funding

The study was supported by the program “*Investissement d'Avenir ANR-10-AIHU 06*” of the French Government, and a grant “*Legs Poix*” from the “*Chancellerie de l'Université de Paris*.” LD was supported by a scholarship from “*Fonds de Dotation Recherche en Santé Respiratoire*” and from “*Fonds d'Etudes et de Recherche des Hôpitaux de Paris*.” LL was supported by *the Institut Universitaire de Cardiologie et Pneumologie de Quebec* (IUCPQ) foundation, Quebec, Canada, a long-term research fellowship from the European Respiratory Society and a post-doctoral research fellowship from the *Fonds de Recherche du Quebec en Santé*.

### Conflict of interest statement

The authors declare that the research was conducted in the absence of any commercial or financial relationships that could be construed as a potential conflict of interest.

## Abbreviations

ANOVA: analysis of variance
VCCO_2_: ventilator-controlled breathing with carbon dioxide stimulation condition
VC: ventilator-controlled breathing condition
VCR: ventilator controlled breathing recovery
CO_2_: carbon dioxide
f: breathing frequency
LEPs: laser-evoked potentials
MDP: multidimensional dyspnea profile
FiCO_2_: inspired fraction of carbon dioxide
PetCO_2_: end-tidal carbon dioxide tension
FB: spontaneous breathing condition
SEPs: somatosensory-evoked potentials
T_I_, inspiratory time, T_E_: expiratory time
T_I_/T_T_: duty cycle
T_T_: total cycle time
${\text{V}}_{\text{E}}^{\prime }$: minute ventilation
VAS: visual analog scale
V_T_: tidal volume
V_T_/T_I_: mean inspiratory flow.
